# Redo aortic arch repair using trifurcated hybrid prosthesis after failed Ascyrus medical dissection stent treatment

**DOI:** 10.1186/s13019-024-03113-y

**Published:** 2024-10-14

**Authors:** Mustafa Al-Obaidi, Razan Salem, Thomas Walther, Tomas Holubec

**Affiliations:** Department of Cardiovascular Surgery, University Heart and Vascular Centre, Goethe University and University Hospital Frankfurt, Frankfurt/Main, Germany

**Keywords:** Acute type a aortic dissection (ATAAD), Ascyrus medical dissection stent (AMDS), Frozen elephant trunk (FET) technique, E-vita OPEN NEO trifurcated hybrid prosthesis, Minimal invasive surgery

## Abstract

**Background:**

The management of acute type A aortic dissection (ATAAD) using the Ascyrus Medical Dissection Stent (AMDS) can lead to complications due to the persistence of the false lumen (FL). This case report presents two instances of failed AMDS treatment for ATAAD, highlighting the novel use of a trifurcated hybrid prosthesis for redo aortic arch repair using a minimally invasive frozen elephant trunk (FET) technique.

**Case Presentation:**

**Case 1**: A 57-year-old male, previously treated with AMDS for ATAAD, presented with an enlarging aortic arch and persistent FL two years post-surgery caused by re-entry in the distal aortic arch. Redo surgery using the FET technique with an E-vita OPEN NEO Trifurcated hybrid prosthesis resulted in successful repair and partial FL thrombosis. **Case 2**: A 51-year-old male with prior AMDS treatment for ATAAD presented with severe aortic valve regurgitation and a maintained FL perfusion due to a residual re-entry in the proximal region of the descending aorta. Redo surgery using the FET technique with the same hybrid prosthesis led to successful repair and good recovery, confirmed by follow-up imaging.

**Conclusions:**

The use of the E-vita OPEN NEO Trifurcated hybrid prosthesis in the FET technique offers a promising solution for redo aortic arch repair in cases of failed AMDS treatment for ATAAD. This approach can improve patient outcomes by addressing complications associated with persistent FL and enhancing long-term survival.

**Supplementary Information:**

The online version contains supplementary material available at 10.1186/s13019-024-03113-y.

## Introduction and background

The Ascyrus medical dissection stent (AMDS; Artivion, Kennesaw, GA), an uncovered stent, enhances the treatment of acute type A aortic dissection (ATAAD; De Bakey I). AMDS is designed to enhance perfusion and facilitate positive remodelling of the dissected aorta [1]. However, there is limited knowledge about late complications and their management [2]. We present two cases highlighting persistent FL with sustained perfusion along with an increased diameter of the distal aortic arch as a complication after treating ATAAD with AMDS and discussing the management challenges with the potential benefits of an early re-operation for improving the long-term survival.

Informed consent for the publication of the study data was obtained from both patients and no institutional review board approval is needed for this report according to the German law.

## Case presentation

### Case 1

**Fig. 1:**
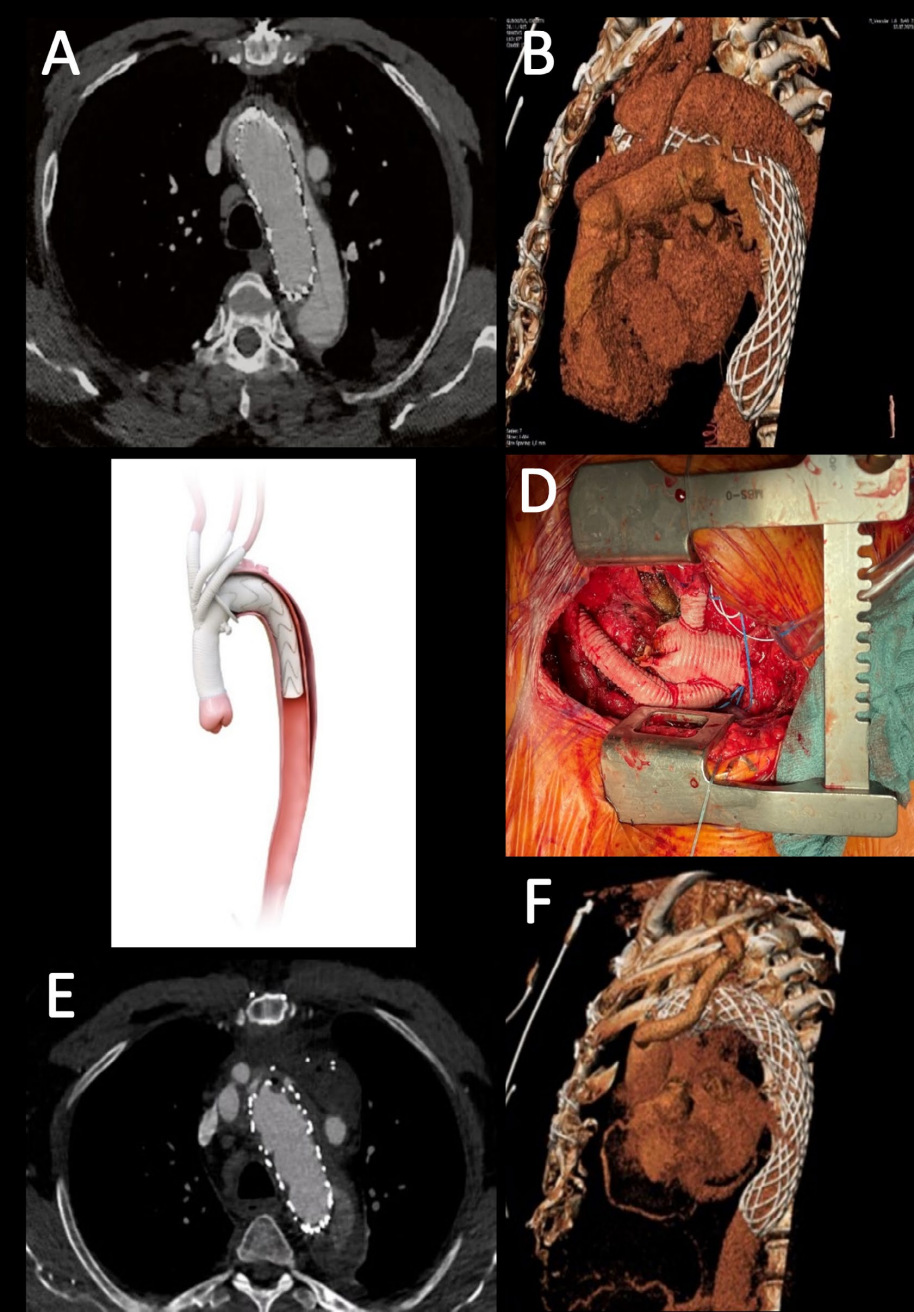
Redo surgery with minimally invasive FET using E-Vita OPEN NEO trifurcated after failed AMDS. (**A**) Preoperative computed tomographic angiography (CTA) revealing an enlarged aortic arch post AMDS implantation with a patent false lumen (FL). (**B**) Preoperative CT 3D reconstruction of AMDS prosthesis. (**C**) The new E-vita OPEN NEO Trifurcated (Permission: Artivion, Inc.). (**D**) Implanted E-vita OPEN NEO trifurcated with debranched supra-aortic vessels via upper ministernotomy. (**E**) Postoperative CTA showing optimal perfusion of supra-aortic vessels and closure of the FL. (**F**) Postoperative CTA 3D reconstruction demonstrating excellent stent-graft implantation and diminished FL diameter

A 57-year-old male patient underwent emergency ascending aorta and aortic arch as well as descending aortic repair with a 55–55 mm AMDS implantation in Zone 0 due to ATAAD (De Bakey I) and debranching of the left subclavian artery (LSA) into the ascending aorta using a 12 mm Dacron prosthesis due to isolated dissection of the LSA at its origin without affecting the aortic arch. The procedure performed at our centre in 2021. Selection and implantation of an appropriate size of AMDS is achieved after an adequate evaluation of a preoperative computed tomographic angiography (CTA) scan and according to the sizing chart and implantation manoeuvre provided by the manufacturer. Two years later, CTA revealed persistent perfusion of the FL throughout the entire aortic arch, descending aorta, brachiocephalic trunk, and left common carotid artery accompanied by significant enlargement of the aortic diameter of 47 mm in the distal aortic arch, along with a collapse of the true lumen (TL) in the descending and abdominal aorta caused by stent-induced new entry (dSINE) tear in the distal aortic arch (Fig. 1A, B). An early redo surgery using a minimally invasive frozen elephant trunk (FET) technique was carried out.

The standard protocol for the minimally invasive aortic arch surgery has previously been described [3]. Briefly, upper ministernotomy to the left 4th intercostal space was performed through a 9-cm-long skin incision and cardio-pulmonary bypass was initiated through the right axillary artery and right femoral vein. Selective LSA perfusion was established with a balloon-inflatable catheter through the 12 mm LSA-prosthesis. After aortic cross-clamping and under cardioplegic arrest the ascending aorta prosthesis was resected 1 cm above the sinotubular junction. By reaching 31 °C body core temperature the left common carotid artery (LCCA) and innominate artery were divided at their origin using vascular staplers (Endo GIA™; Medtronic/Covidien, Minneapolis, MN), followed by LCCA intubation with a balloon catheter, achieving trilateral antegrade cerebral perfusion (ACP). At 28 °C temperature the cross-clamp was removed, and the ascending aortic prosthesis was resected up to the suture felt ring of AMDS (zone 0) followed by AMDS suture felt ring dilatation using 30 mm Hegar dilator. Subsequently, the hybrid prosthesis was implanted in the FET technique (E-vita OPEN NEO Trifurcated 30/30 mm, and 180 mm length of the stent-graft (Artivion/JOTEC, Kennesaw, GA) (Fig. 1C). The appropriate size of the prosthesis was selected and implanted in regard to the anatomic features of the aorta along with the preoperative CTA-scan evaluation and measurements according to the sizing chart and implantation procedure provided by the manufacturer [3] (Video). Followed by anastomosis of all three supra-aortic vessels and proximal aortic anastomosis (Fig. 1D and Video). The total time of cardio-pulmonary bypass, aortic cross-clamp and cerebral perfusion was 160 min, 88 min, and 26 min, respectively.

The patient was extubated 4 h after surgery and the postoperative course was uneventful. Control CTA after 1 week demonstrated a perfectly implanted stent-graft as well as partial thrombosis of the FL (Fig. 1E, F).

### Case 2

**Fig. 2:**
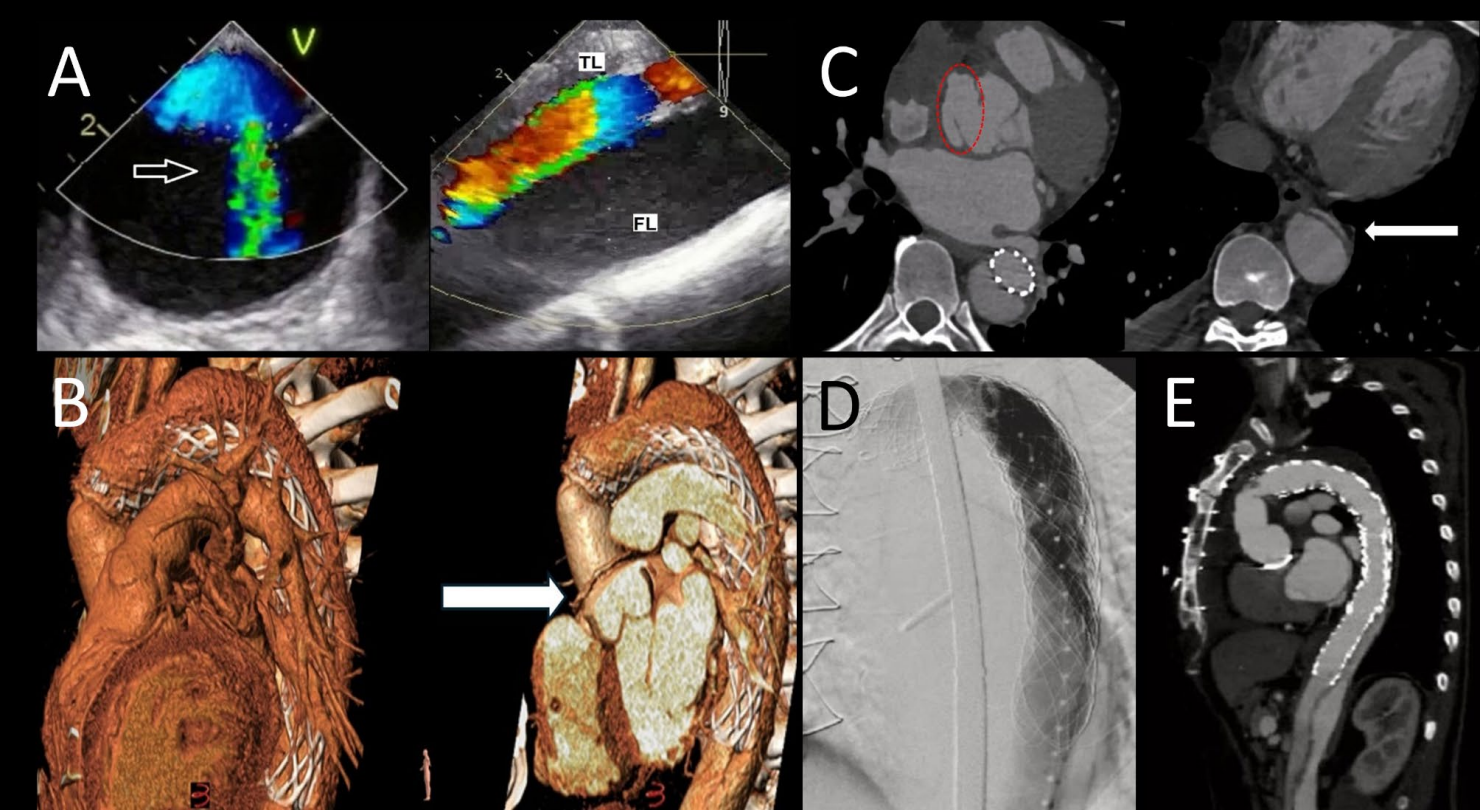
FET using E-Vita OPEN NEO trifurcated after failed AMDS as a redo surgery via re-sternotomy. (**A**) Transoesophageal echocardiography of descending aorta demonstrating a true lumen (TL) and an enlarged false lumen (FL) with maintained perfusion through re-entry (arrow). (**B**) Computer tomography (CT) 3D reconstruction showing aortic root pseudoaneurysm (arrow). (**C**) CT angiography (CTA) cross-sectional revealing pseudoaneurysm at aortic root (red circle) and subtotal true lumen collapse (arrow) at the descending aorta. (**D**) Intraoperative fluoroscopy displaying excellent E-Vita OPEN NEO Trifurcated implantation with stent-graft extension. (**E**) CTA control after 6 months showing patent aortic arch, E-Vita OPEN NEO with stent-graft extension, and obliterated FL with partial thrombosis

A 51-year-old male patient presented with ATAAD (De Bakey I) in 2021 and received an emergency aortic valve (AV) and root reconstruction. This was performed in the setting of obliteration of the proximal FL down to the level of the aortic annulus, along with resuspension of the aortic valve commissures incorporated into the aortic root reconstruction. Additionally, the patient received ascending aorta and aortic arch repair with a 40–40 mm AMDS implantation. One year later, he presented with acute progressive dyspnoea due to cardiac decompensation. Transoesophageal echocardiography revealed severe AV regurgitation and maintained FL perfusion due to a residual re-entry in the proximal region of the descending aorta (Fig. 2A). CTA also revealed a saccular pseudoaneurysm arising from the aortic root (Fig. 2B, C) and a near TL collapse of the descending aorta (Fig. 2C).

An urgent redo operation was performed via re-sternotomy comprising total arch replacement (TAR) in the FET technique using the trifurcated E-vita OPEN NEO Trifurcated 26/24 mm, and 180 mm length of the stent-graft (Fig. 1C). In addition, the patient received an aortic valve and aortic root replacement using a 25 mm mechanical conduit prosthesis (Masters HP Series; SJM/Abbott; Santa Clara; USA), with reimplantation of coronary arteries (Bentall-de Bono procedure). The total time of cardio-pulmonary bypass, aortic cross-clamp and cerebral perfusion was 220 min, 146 min, and 24 min, respectively.

Postoperative CTA prompted a distal extension of the FET stent through thoracic endovascular aortic repair (TEVAR) (Fig. 2D), resulting in a good recovery. The patient was discharged on the 29th postoperative day. A routine 6 month follow-up CTA showed optimal results with a perfectly implanted stent-graft, and partial thrombosis of the FL (Fig. 2E).

## Discussion

The standard repair for ATAAD involves resection of the primary tear and closure of the FL at the distal anastomosis. This manuscript explores cases where AMDS fails to close the FL, either due to a new entry at the distal anastomosis postoperatively or initial difficulties in resecting the distal tear, both contributing to persistent FL perfusion and malperfusion management failure [4].

Compared to hemiarch replacement with AMDS, the TAR with FET technique offers the advantage of placing a stent-graft into the TL of the distal arch, sealing re-entry tears in both the aortic arch and proximal descending thoracic aorta in a single-stage procedure, as demonstrated in case (1) Along with the Z-shape stent design of the E-Vita Open Neo Trifurcated prosthesis could reduce the risk of dSINE as it exerts less focal stress to the aortic wall. Additionally, it creates a covered landing zone for a second-stage TEVAR in case of persistent FL perfusion, as shown in case (2) However, this approach can increase the time of both cardio-pulmonary bypass and circulatory arrest increasing the risk of stroke or haemorrhage [5] .

The DARTS trial [1] reported successful AMDS implantation in 46 patients, with a 3-year follow-up revealing an increase in the aortic diameter in zones 1 and 2 in 27.3% and 25% of patients, respectively. Additionally, a patent FL was observed in 40% of patients in zone 1 and 31.8% in zone 2. Six patients (13%) required disease-related reinterventions; however, no reinterventions on the aortic arch were necessary.

Montagner et al. [6] observed that the low radial force of the AMDS stent is primarily designed to realign the intimal layer against the media and adventitia. The ultimate resolution of malperfusion relies on the successive expansion of the TL. In acute aortic dissection, the intimal flap remains highly mobile, allowing the distal end of the stent to expand fully, positioning the dissection flap adjacent to the outer aortic wall [7]. However, the emergence of new entry tears may keep the FL patent, causing further expansion of the FL at the expense of the low radial force of the AMDS, as evidenced in both cases.

The AMDS lacks sufficient radial force to reliably ensure the distal expansion of the TL, especially in cases of visceral malperfusion, as reported by Kanj et al. [8]. Importantly, this limited radial force should not prompt surgeons to oversize the stent, as this could result in stent collapse due to the higher radial force exerted by the aortic arch. Moreover, excessive radial force on the aortic arch wall may result in dSINE, as shown in case 1.

The radial force of endovascular stents plays a critical role in supporting blood vessels, maintaining lumen patency, and ensuring secure fixation to the arterial wall [9]. Radial force varies between stent designs, influenced by factors such as stent type, deployment site, and the specific characteristics of each stent layer [10]. Given this variability, oversizing the AMDS should be avoided, and adherence to manufacturer-recommended sizes is strongly advised.

While the AMDS has potential, its ability to promote positive aortic remodelling remains uncertain and appears less likely when compared to the FET technique. The primary issue seems to lie in the AMDS’s insufficient radial force, which tends to elongate rather than expand, limiting its effectiveness in this regard [8].

Another notable characteristic of the AMDS is that it is an uncovered stent. The main limitation of bare stents is their inability to seal entry tears, unlike covered grafts that can immediately close tears while preserving the patency of the vessel. In this scenario, any entry tear not sealed by the bare stent will remain active. Therefore, the use of AMDS should be reserved for type A aortic dissections where the primary tear is located in the root or ascending aorta, ensuring the aortic arch is free of any additional tears for optimal results.

In the rare instances where aortic arch reintervention is necessary in zones covered by AMDS, significant challenges arise due to the potential ingrowth of the stent into the aortic wall. Removal of the stent poses a high risk of damaging the aortic wall, potentially causing rupture. Cutting the AMDS can lead to stent unwinding, increasing the risk of late stent migration and associated mortality. An alternative approach, such as aortic arch debranching followed by endovascular stent grafting, could be considered, however, the recoil properties of AMDS complicate efforts to seal the aorta using endovascular solutions.

The outlined strategy facilitates the repair by keeping the AMDS in place, allowing the FET technique in aortic zone 0 or 1 with debranching of head vessels. Moreover, it provides a secure landing zone for TEVAR in cases of persistent FL perfusion.

## Conclusion and central message

The introduction of the E-Vita OPEN NEO Trifurcated aortic prosthesis has revolutionized TAR procedures by simplifying complexity. Surgeons can now employ the FET technique in aortic zone 0 or 1, contrasting with the traditional zone 2 or 3, allowing for the placement of a covered stent that seals re-entries in the aortic arch and proximal descending aorta. Additionally, it provides a secure landing zone for TEVAR in cases of persistent FL perfusion caused by distal re-entry [11].

This advancement not only streamlines the procedure but also opens the possibility of a minimally invasive approach in re-do aortic surgeries, as highlighted by the senior author of this manuscript in their publication on the E-Vita OPEN NEO Trifurcated system [3].

Total arch repair in FET using E-vita OPEN NEO Trifurcated hybrid prosthesis is an excellent option for redo surgical treatment of failed AMDS surgical treatment for acute De Bakey I aortic dissection.

## Electronic supplementary material

Below is the link to the electronic supplementary material.

Supplementary Material 1

## Data Availability

No datasets were generated or analysed during the current study.

## Abbreviations

ATAAD: Acute type A aortic dissection
AMDS: Ascyrus Medical Dissection Stent
FL: False lumen
FET: Frozen elephant trunk
LSA: Left subclavian artery
CTA: Computed tomographic angiography
TL: True lumen
dSINE: Distal stent-induced new entry
LCCA: Left common carotid artery
ACP: Antegrade cerebral perfusion
AV: Aortic valve
TAR: Total arch replacement
TEVAR: Thoracic endovascular aortic repair
